# CheV enhances the virulence of *Salmonella Enteritidis*, and the *Chev*-deleted *Salmonella* vaccine provides immunity in mice

**DOI:** 10.1186/s12917-024-03951-x

**Published:** 2024-03-11

**Authors:** Lu Zhang, Tonglei Wu, Fengjie Wang, Wan Liu, Guixin Zhao, Yanying Zhang, Zhiqiang Zhang, Qiumei Shi

**Affiliations:** Hebei Key Laboratory of Preventive Veterinary Medicine, College of Animal Science and Technology, Hebei Normal University of Science & Technology, Qinhuangdao, 066004 PR China

**Keywords:** *Salmonella enteritidis*, *cheV*, Gene deletion, Virulence, Vaccine

## Abstract

**Background:**

*Salmonella enteritidis* (*SE*) is a major zoonotic pathogen and causes infections in a variety of hosts. The development of novel vaccines for *SE* is necessary to eradicate this pathogen. Genetically engineered attenuated live vaccines are more immunogenic and safer. Thus, to develop a live attenuated *Salmonella* vaccine, we constructed a *cheV* gene deletion strain of *SE* (named Δ*cheV*) and investigated the role of *cheV* in the virulence of *SE*. First, the ability to resist environmental stress in vitro, biofilm formation capacity, drug resistance and motility of Δ*cheV* were analyzed. Secondly, the bacterial adhesion, invasion, intracellular survival assays were performed by cell model. Using a mouse infection model, an in vivo virulence assessment was conducted. To further evaluate the mechanisms implicated by the reduced virulence, qPCR analysis was utilized to examine the expression of the strain’s major virulence genes. Finally, the immune protection rate of Δ*cheV* was evaluated using a mouse model.

**Results:**

Compared to C50336, the Δ*cheV* had significantly reduced survival ability under acidic, alkaline and thermal stress conditions, but there was no significant difference in survival under oxidative stress conditions. There was also no significant change in biofilm formation ability, drug resistance and motility. It was found that the adhesion ability of Δ*cheV* to Caco-2 cells remained unchanged, but the invasion ability and survival rate in RAW264.7 cells were significantly reduced. The challenge assay results showed that the LD_50_ values of C50336 and Δ*cheV* were 6.3 × 10^5^ CFU and 1.25 × 10^7^ CFU, respectively. After the deletion of the *cheV* gene, the expression levels of *fimD*, *flgG*, *csgA*, *csgD*, *hflK*, *lrp*, *sipA*, *sipB*, *pipB*, *invH*, *mgtC*, *sodC*, *rfbH*, *xthA* and *mrr1* genes were significantly reduced. The live attenuated Δ*cheV* provided 100% protection in mice against *SE* infection.

**Conclusion:**

All the results confirmed that the deletion of the *cheV* gene reduces the virulence of *SE* and provides significant immune protection in mice, indicating that Δ*cheV* could be potential candidates to be explored as live-attenuated vaccines.

**Supplementary Information:**

The online version contains supplementary material available at 10.1186/s12917-024-03951-x.

## Background

*Salmonella Enteritidis* (*SE*) ranks as a major zoonotic pathogen, prevalent in both livestock, poultry wildlife and humans. It leads to considerable economic losses in animal agriculture and is responsible for numerous human fatalities. This pathogen poses a severe threat to both economic progress and public health [1]. *SE* infection in humans is mainly transmitted through the environment, animal carriers, and water. Poultry and livestock, as food-producing animals, are considered the primary sources of human *SE* infections [2].

For many years, using antibiotics was the primary method for controlling *Salmonella* infection. However, the development of multiple antibiotic resistance mechanisms in bacteria has led to a reevaluation of antibiotic use. Currently, vaccination is considered the most effective and cost-efficient method to reduce or control *Salmonella* infection [3]. *Salmonella* vaccines commercially available are typically classified into three main types: live attenuated vaccines, inactivated vaccines, and subunit vaccines. The immunological effects of these vaccines vary significantly, as established by numerous studies. While inactivated vaccines are known for their safety and ability to elicit high antibody levels, they often fall short in providing adequate immune protection. This shortfall is attributed to their lack of ability to induce robust cell-mediated immunity, which is essential for the eradication of intracellular pathogens like *Salmonella* [4, 5]. On the other hand, subunit vaccines are also safe but usually necessitate multiple doses to extend the duration of immunity, and require suitable adjuvants to elicit a potent cellular immune response. Genetically the live attenuated vaccines, created by knocking out virulence genes, can induce strong cell-mediated immunity, because the vaccine strain can invade the host and present nearly all antigenic components of the bacteria [6–8]. Identifying virulence genes is key to developing gene deletion live vaccines, and many researchers have already developed a variety of vaccines using this method, including for *Brucella*, *Salmonella Typhimurium*, *Yersinia pestis*, *Vibrio anguillarum*, and *Edwardsiella tarda* [9–13].

*SE* spreads through the fecal-oral route and, to successfully colonize the host, must overcome the acidic stress of the gastrointestinal tract, adhere to the intestinal mucosa, and disrupt epithelial cells. It can also penetrate beneath the mucosa to be phagocytosed by macrophages. As an intracellular parasite, *SE* has evolved remarkable capabilities to resist intracellular bactericidal components. It then spreads throughout the body via the bloodstream or lymphatic system, carried by macrophages, leading to systemic infection [14]. The chemotaxis allows *Salmonella* to perceive changes in the external environment within host tissues, such as pH, osmotic pressure and temperature, and evade host immune pressure, overcome colonization resistance and resist antimicrobial treatment by regulating the expression of specific genes, thus surviving and colonizing within the host. Additionally, the chemotaxis also controls drug resistance, biofilm formation and virulence [15–18]. Researches indicate that CheV is a crucial chemotactic protein closely associated with various pathogenic processes. These include the adhesion and chemotaxis in *Vibrio cholerae*, chemotaxis in *Bacillus subtilis*, biofilm formation and motility in *Campylobacter jejuni* and *Vibrio anguillarum*, the adhesion, chemotaxis, and motility of *Vibrio harveyi*, as well as the colonization and infection processes in *Helicobacter pylori* [19–22]. Genomic analysis has revealed that *Salmonella Enteritidis* (*SE*) carries the *cheV* gene. Researchers using differential proteomics analysis on *Salmonella Typhimurium* found that CheV expression decreases after infecting epithelial cells, suggesting that this gene may influence the virulence of SE through multiple mechanisms [23].

Based on these studies, we hypothesize that the *cheV* gene plays an important role in *SE*’s biofilm formation, adhesion, invasion ability and virulence. To test this hypothesis, we constructed a *cheV* gene deletion strain using homologous recombination technology and analyzed the impact of the *cheV* gene on the virulence of *SE* through in vitro stress simulation tests, detection of biofilm formation ability, drug resistance, motility, adhesion, invasion, intracellular survival assays, LD_50_ determination and virulence gene expression analysis. Additionally, to demonstrate the feasibility of using the *cheV* gene deletion strain as a vaccine against *Salmonella*, we evaluated the immune protection rate of Δ*cheV* using a mouse model. These study results provide an important theoretical basis for understanding the impact of the *cheV* gene on *SE*’s virulence.

## Methods

### Bacterial strains, cells and plasmids

Bacterial strains and plasmids used in this study are listed in Table 1. *Salmonella enteritidis* C50336 was maintained in the Key Laboratory of Preventive Veterinary Medicine, Hebei Province. The bacteria are grown in Luria-Bertani (LB) broth (Haibo Biotechnology Co., Ltd., China) at 37 °C, unless otherwise specified. The Caco-2 BBE cells and RAW264.7 cells used in this study were provided by BeNa Culture Collection (Shanghai, China). Both types of cells are cultured in DMEM (Thermo Fisher Scientific Co., Ltd., China) containing 10% fetal bovine serum (Thermo Fisher Scientific Co., Ltd., China), add antibiotics when necessary, such as 50 µg/mL streptomycin and 50 U/mL penicillin, or 50 µg/mL gentamicin, in an incubator with 5% CO_2_.

**Table 1:** Bacterial strains and plasmids used in this study

Strains	Relevant characteristics	Sources
C50336	Wild-type *S. enteritidis*	This study
Δ*cheV::cat*	a first recombination strain	This study
Δ*cheV*	a second recombination strain	This study
Δ*cheV* + *cheV*	Δ*cheV*-complemented strain	This study
**Plasmids**	**Characteristics**	**Sources**
pKD3	FRT-Cm-FRT cassette (Cb^R^ Cm^R^)	invitrigen
pKD46	Apr, containing the Red recombinase of λ phage	invitrigen
pCP20	FLP recombinase, temperature sensitive replication (AM^R^)	invitrigen

### Experimental animals

Kunming mice were purchased from Beijing Speifu Biotechnology Co., Ltd. (Beijing, China). Female Kunming mice aged 4–6 weeks were used for LD_50_ testing, and female Kunming mice aged 6–8 weeks were used for immune protection testing.

### Construction of the *cheV* gene deletion strain

The *cheV* gene deletion strain was constructed using the λ-Red recombination technique [24]. The C50336 strain carrying the pKD46 plasmid was cultured in LBA containing 225 mg/mL L-arabinose at 30 °C until its OD_600 nm_ reached 0.6–0.8. It was then washed 3 times with pre-cooled autoclaved ultrapure water and 10% glycerol to prepare competent cells. Next, using the pKD3 plasmid as a template, specific primers P1, P2 (Table 2) were used to amplify homologous fragments. The purified fragments were electroporated into C50336 (pKD46) competent cells, followed by the addition of 1 mL of LB liquid medium and incubation at 30 °C for 2 h. The culture was then spread on LB agar containing 50 µg/mL chloramphenicol (Cm). Colonies that grew were identified using specific primers P3, P4 (Table 2). Positive strains, after removal of the pKD46 plasmid at 42 °C, were named Δ*cheV::cat*. To remove the Cm segment, the pCP20 plasmid was electroporated into Δ*cheV::cat*, following the same process, and mutants were identified using specific primers P3, P4. The positive strains, after removal of the pCP20 plasmid at 42 °C, were named Δ*cheV*.

To generate the Δ*cheV*-complemented strain, polymerase chain reaction (PCR) was performed with the primers P5, P6 (Table 2) to amplify the *cheV* open reading frame. The purified PCR product was then chemically transformed into PMD-19T vector. The PMD-19T-*cheV* was electroporated into Δ*cheV* and P5, P6 was used to confirm the complemented strain. The positive strains were named Δ*cheV* + *cheV.*

**Table 2:** Primers used for constructing the mutant and the complemented strain

Primer	Sequence (5’ − 3’)
P1:	TGGGCATGGTCAATATTCGGGATCAGGTCATTCCGGTGATTGATTTGCCAGCGGTAGCGTGTGTAGGCTGGAGCTGCTTCG
P2:	TCATTGGCGCTGCCGGAAAGCGAGGAGTGGATAACGACCGGGATCTTTTTCAGCCGTTCCATATGAATATCCTCCTTAG
P3:	TGAACTGCTGTTATTCCGTCTTGG
P4:	ATCAGTGGTCCCTGGCTGTTTG
P5:	GTTAGGATGGACAATTTTCAGAAAGATA
P6:	TCAGGCGCTTTTGCGGCTGATCAGTGGT

### In vitro stress simulation assay

The bacterial culture during the logarithmic phase was washed 3 times with sterilized saline, and then serially diluted for counting, to determine the original bacterial count. The culture was incubated separately in strong acid solution (pH 3.5), strong alkaline solution (pH 10), at 42 °C for 1 h and in an oxidative stress solution (10 mmol/L H_2_O_2_) for 10 min. The bacteria were counted again after these stress treatments to determine the post-stress bacterial count. The survival rate was calculated as the post-stress bacterial count divided by the original bacterial count.

### Detection of biofilm formation

Referring to previous research methods, the ability to form biofilms was assessed using the crystal violet (CV) staining method [25]. C50336, Δ*cheV* and Δ*cheV* + *cheV* were inoculated into 6 mL of LB liquid medium (Δ*cheV* + *cheV* was in LBA) and incubated statically at 28 °C for 3 days. The biofilms were washed with PBS, fixed in absolute methanol for 15 min, and stained with 2% CV for 15 min to observe the thickness and staining of the bacterial ring. The quantitative detection method is as follows: 150 µL of bacterial suspension was added to each well of a 96-well plate, stained as above, and finally, 200 µL of absolute ethanol was added to each well to dissolve the CV. The absorbance was measured at 570 nm, and the assay was repeated 3 times.

We also studied the effect of the *cheV* gene on the main components of biofilms: curli and cellulose formation. Referring to previous methods [26], 5 µL of overnight-cultured bacterial suspension was inoculated onto LB agar containing 160 mg/L Congo red and 10 mg/L Coomassie brilliant blue without salt, incubated at 28 °C for 2 days, and the colony morphology and color were observed to assess the production of curli. Similarly, 5 µL of overnight-cultured bacterial suspension was inoculated onto LB agar without salt containing Calcofluor White Stain (200 mg/L) and incubated at 28 °C for 2 days. The production of cellulose was evaluated by observing the fluorescence intensity under ultraviolet (UV) light (366 nm).

### Detection of drug resistance

The sensitivity of Δ*cheV* to antibiotics was tested using the Kirby-Bauer (K-B) method. According to the standards of the United States National Committee for Clinical Laboratory Standards (NCCLS), 17 drugs were selected for testing. These drugs include: ampicillin, piperacillin, amoxicillin, cefazolin, cephalothin, cefoperazone, cefuroxime, ceftriaxone, gentamicin, amikacin, kanamycin, streptomycin, tetracycline, doxycycline, minocycline, ciprofloxacin and levofloxacin. Overnight-cultured bacterial suspension was evenly spread on LB agar plates. Commercial antibiotic susceptibility disks were placed on the inoculated plates using tweezers. The plates were then incubated at 37 °C for 12 h, and the diameters of the inhibition zones were measured. The results were interpreted according to the NCCLS antibiotic susceptibility testing standards.

### Motility assay

Referring to previous methods [27, 28], 5 µL of overnight-cultured bacterial suspension was inoculated onto LB semi-solid agar plates (0.3% agar). The plates were then incubated at 37 °C for 5–6 h. The diameter of the swimming halo was measured, and the assay was repeated 3 times.

### Adhesion, invasion and intracellular survival assays

As previously described [9, 25], Caco-2 cells were seeded at a density of 1 × 10^5^ cells per well in a 12-well plate. C50336, Δ*cheV* and Δ*cheV* + *cheV* were resuspended in PBS for counting and added to the cells with a multiplicity of infection (MOI) of 100. The mixture was centrifuged at 1000 rpm for 5 min and then incubated in a 37 °C, 5% CO_2_ incubator for 1 h. The cells were lysed with 1% Triton X-100 for 8 min, and the lysates were serially diluted for counting. For the invasion assay, after infecting the cells as described above, the cells were further incubated in DMEM containing gentamicin (100 µg/mL) for 1 h to remove extracellular bacteria. The cells were then lysed with 1% Triton X-100 and counted. Adhesion rate = (number of adherent bacteria) / (number of infecting bacteria per well) × 100%; Invasion rate = (number of invaded bacteria) / (number of infecting bacteria per well) × 100%.

For the intracellular survival assay, as previously described [25, 28, 29], RAW264.7 cells were seeded at a density of 10^5^ cells per well in 2–12 well plates. C50336, Δ*cheV* and Δ*cheV* + *cheV* infected the cells with an MOI of 100 and were incubated in a 37 °C, 5% CO_2_ incubator for 2 h. The cells were then washed twice with PBS to remove non-adherent/invasive bacteria. Subsequently, the cells were incubated in DMEM containing gentamicin (100 µg/mL) for 1 h to eliminate all remaining extracellular bacteria. The cells were lysed with 1% Triton X-100 and counted as the 3-h intracellular bacterial count. Cells in the other plate were incubated in DMEM containing 10 µg/mL gentamicin for 20 h, lysed with 1% Triton X-100, and counted as the 23-h intracellular bacterial count. Intracellular survival rate = (number of intracellular bacteria at 23 h) / (number of intracellular bacteria at 3 h) × 100%.

### Determination of LD_50_ in mice

Referring to previous methods [9], 55 female Kunming mice, aged 4–6 weeks, were randomly divided into 11 groups (*n* = 5). The first five groups were intraperitoneally (i.p.) injected with C50336 at doses ranging from 2 × 10^7^ to 2 × 10^3^ CFU/mouse. The next five groups were i.p. injected with Δ*cheV* at doses ranging from 2.5 × 10^9^ to 2.5 × 10^5^ CFU/mouse. The remaining group was i.p. injected with an equal volume of PBS. The death of mice was observed and recorded over a period of 14 days. The LD_50_ value was calculated using the formula of log_10_ [50% endpoint] = A + (B × C), where A = log_10_ [infectious dose showing a mortality next below 50%], B = difference of logarithms = [50% – (mortality at infectious dose next below 50%)] / [(mortality next above 50%) – (mortality next below 50%)], and C = log_10_ [difference between serial infectious doses used in challenge studies] [30].

### RNA extraction and quantitative real-time PCR (qPCR)

To further investigate the role of the *cheV* gene in the virulence of *SE*, we employed qPCR technology to detect the expression levels of virulence genes. RNA was extracted using a bacterial RNA extraction kit (Beijing Aidlab Biotechnologies Co., Ltd., China), and DNA was removed through DNase I treatment. Then, RNA was reverse-transcribed into cDNA using a reverse transcription kit (Bohang Biotechnology Co., Ltd., China). Using this cDNA as a template and based on literature [31, 32], primers were designed (Table 3). qPCR detection was performed using the SYBR Green dye method.

**Table 3:** Primers used for qPCR

Gene	Sequence (5’ − 3’)	
*fimD*-F	CGCGGCGAAAGTTATTTCAA	
*fimD*-R	CCACGGACGCGGTATCC	
*flgG*-F	GCGCCGGACGATTGC	
*flgG*-R	CCGGGCTGGAAAGCATT	
*prot6E*-F	GAACGTTTGGCTGCCTATGG	
*prot6E*-R	CGCAGTGACTGGCATCAAGA	
*csgA*-F	AATGCCACCATCGACCAGTG	
*csgA*-R	CAAAACCAACCTGACGCACC	
*csgD*-F	GCCTCATATTAACGGCGTG	
*csgD*-R	AGCGGTAATTTCCTGAGTGC	
*bcsA*-F	GCCCAGCTTCAGAATATCCA	
*bcsA*-R	TGGAAGGGCAGAAAGTGAAT	
*ompR*-F	TGTGCCGGATCTTCTTCCA	
*ompR*-R	CTCCATCGACGTCCAGATCTC	
*hflK*-F	AGCGCGGCGTTGTGA	
*hflK*-R	TCAGACCTGGCTCTACCAGATG	
*tatA*-F	AGTATTTGGCAGTTGTTGATTGTTG	
*tatA*-R	ACCGATGGAACCGAGTTTTTT	
*lrp*-F	TTAATGCCGCCGTGCAA	
*lrp*-R	GCCGGAAACCAAATGACACT	
*sipA*-F	CAGGGAACGGTGTGGAGGTA	
*sipA*-R	AGACGTTTTTGGGTGTGATACGT	
*sipB*-F	GCCACTGCTGAATCTGATCCA	
*sipB*-R	CGAGGCGCTTGCTGATTT	
*pipB-*F	GCTCCTGTTAATGATTTCGCTAAAG	
*pipB-*R	GCTCAGACTTAACTGACACCAAACTAA	
*invH-*F	CCCTTCCTCCGTGAGCAAA	
*invH-*R	TGGCCAGTTGCTCTTTCTGA	
*mgtC*-F		CGAACCTCGCTTTCATCTTCTT
*mgtC*-R		CCGCCGAGGGAGAAAAAC
*sodC*-F	CACATGGATCATGAGCGCTTT	
*sodC*-R	CTGCGCCGCGTCTGA	
*orf245*-F	CAGGGTAATATCGATGTGGACTACA	
*orf245*-R	GCGGTATGTGGAAAACGAGTTT	
*rfbH-*F	ACGGTCGGTATTTGTCAACTCA	
*rfbH-*R	TCGCCAACCGTATTTTGCTAA	
*xthA*-F	CGCCCGTCCCCATCA	
*xthA*-R	CACATCGGGCTGGTGTTTT	
*mrr1*-F	CCATCGCTTCCAGCAACTG	
*mrr1*-R	TCTCTACCATGAACCCGTACAAATT	
*16 S rRNA*-F *16 S rRNA*-R	CCAGGGCTACACACGTGCTA TCTCGCGAGGTCGCTTCT	

### Immunization of Δ*cheV* via i.p. route

Referring to previous methods [8], 30 female Kunming mice, aged 6–8 weeks, were randomly divided into 3 groups (*n* = 10), namely the immune group (Group A), the C50336 infection group (Group B) and the PBS control group (Group C). Group A was i.p. injected with a dose of 2.5 × 10^6^ CFU/mouse of Δ*cheV* (once on day 0 and once on day 14), while Group B and C were i.p. injected with an equal volume of PBS. 45 days after the second immunization, mice in Groups A and B were simultaneously i.p. injected with a dose of 2 × 10^7^ CFU/mouse of C50336, and Group C was injected with an equal volume of PBS. The survival of mice was recorded daily for 14 days post-infection (dpi). The immune protection rate of Δ*cheV* to mice can be calculated according to the formula: (mortality rate of the control group - mortality rate of the immune group)/mortality rate of the control group × 100%.

### Statistical analysis

Statistical analyses were conducted using GraphPad Prism v 8.0, employing one-way Analysis of Variance (ANOVA) followed by t-tests. Data are presented as mean ± standard error. Significant differences are indicated with an asterisk (*), where *p < 0.05, **p < 0.01, ***p < 0.001 are considered to represent statistically significant differences in mean values.

## Results

### The *cheV* gene affects the resistance of *SE* to environmental stress

**Fig. 1:**
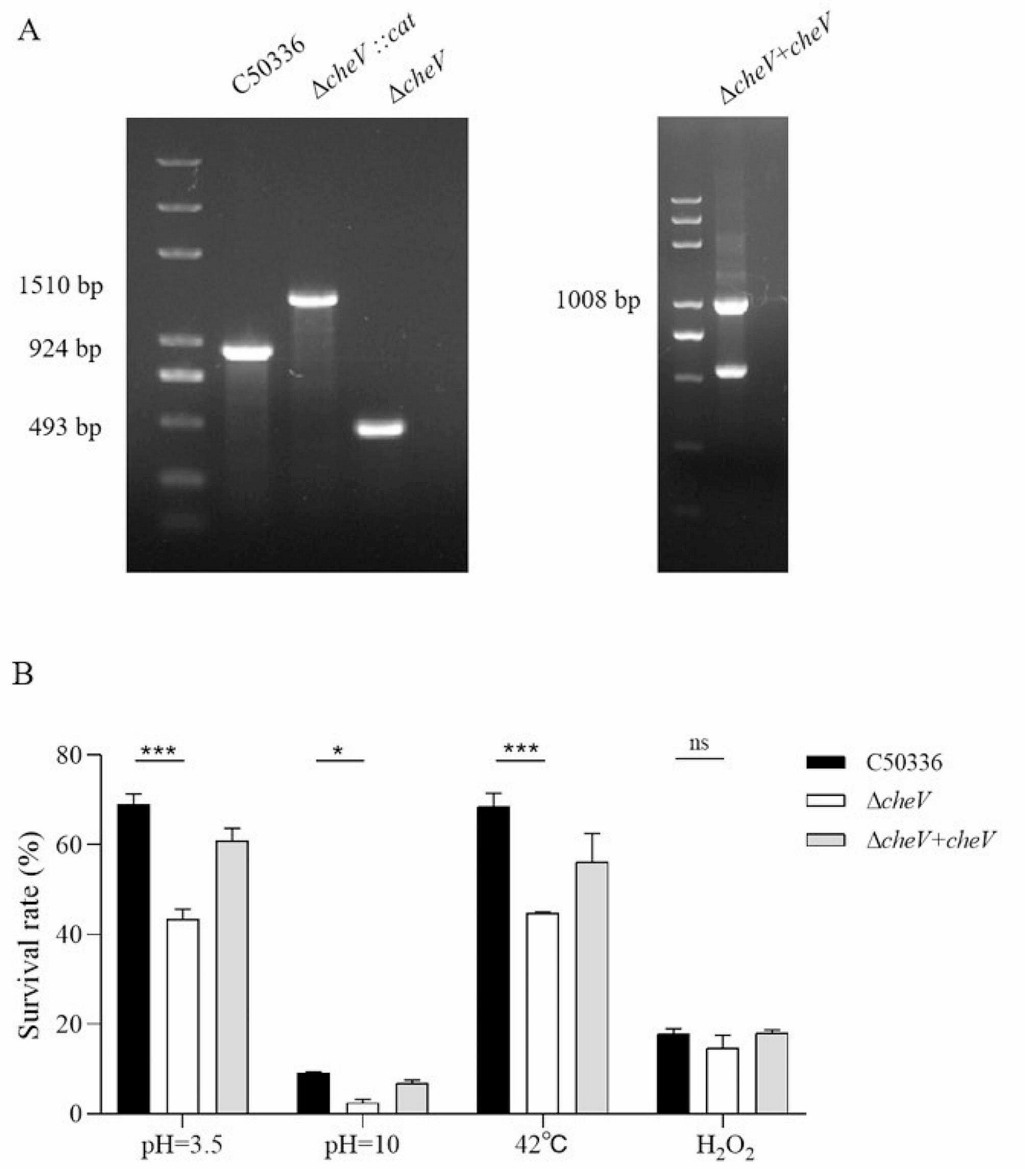
(**A**) PCR verification of the *cheV* gene deletion strain and Δ*cheV*-complemented strain. C50336 means the wild-type strain; Δ*cheV::cat* means a first recombination strain; Δ*cheV* means a second recombination strain, Δ*cheV* + *cheV* means Δ*cheV*‐complemented strain. The PCR product of C50336 has a length of 924 bp, the product of Δ*cheV::cat* has a length of 1510 bp, the product of Δ*cheV* has a length of 493 bp and the product of Δ*cheV* + *cheV* has a length of 1008 bp. (**B**) The survival rate of Δ*cheV* under various environmental stresses. The data represents the average of 3 replicates (*p < 0.05, ***p < 0.001, ns means not significant)

Using λ-Red recombination technology, a *cheV* gene deletion mutant was constructed in C50336. As shown in Fig. 1A, the deletion strain of the *cheV* gene and the complementation strain were successfully constructed.

To study whether the *cheV* gene influences the resistance of *SE* to various environmental stresses, we compared the survival of C50336 and Δ*cheV* in strong acid stress solution, strong alkaline stress solution, at 42 °C and in oxidative stress solution. The results (Fig. 1B) showed that, compared to C50336, the survival rate of the Δ*cheV* significantly decreased in acidic, alkaline and thermal environments. There was no significant change in survival rate under oxidative stress conditions. This indicates that the *cheV* gene plays a role in affecting the resistance of *SE* to acid, alkaline and thermal stresses.

### The *cheV* gene does not affect biofilm formation ability and drug resistance of *SE*

**Fig. 2:**
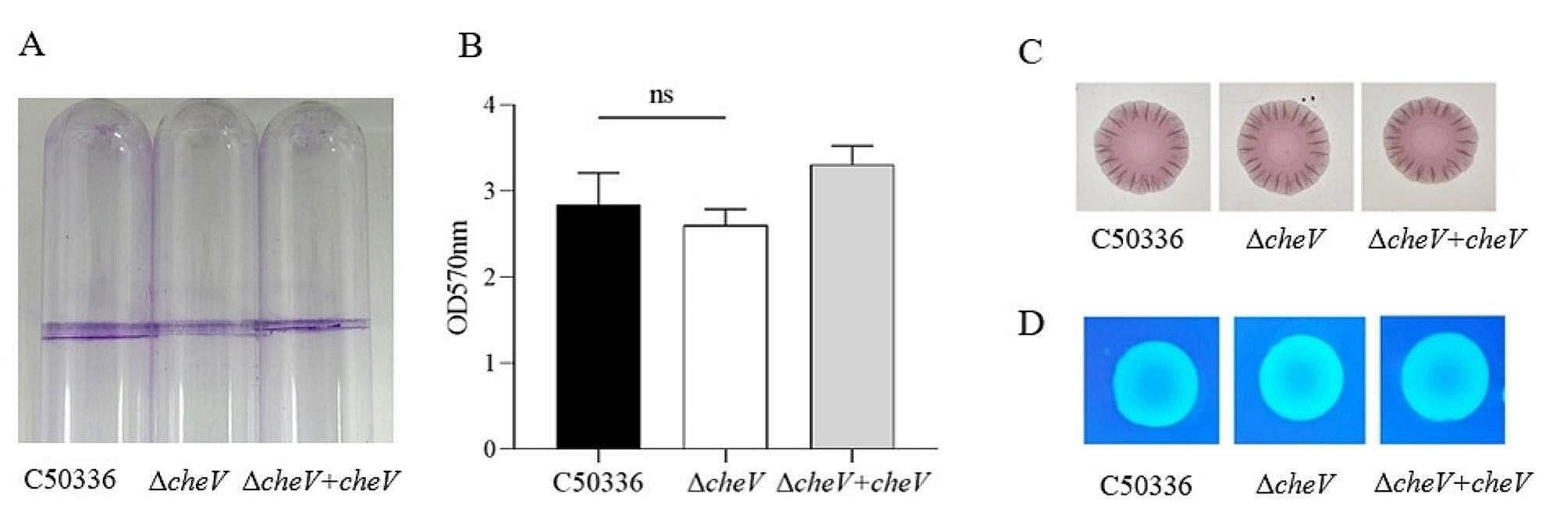
(**A**) Detection of biofilm formation in glass test tubes. (**B**) Qualitative detection of biofilm formation in microtiter plates, with absorbance measured at 570 nm (ns means not significant). (**C**) Curli formation detection. (**D**) Cellulose formation detection

Biofilm formation assays were conducted for C50336, Δ*cheV* and Δ*cheV + cheV*. The results showed that in test tubes, C50336, Δ*cheV* and Δ*cheV* + *cheV* were all capable of forming biofilms (Fig. 2A), with no significant differences in thickness and color intensity. Quantitative results revealed that the biofilms formed by the 3 strains had similar OD_570 nm_ values after staining and dissolution (Fig. 2B). Curli detection indicated that all 3 strains formed red, rough colonies (Fig. 2C), suggesting that all strains could produce curli. The cellulose detection results showed that the colonies of all 3 strains emitted the same fluorescence intensity under UV light (Fig. 2D). These findings suggest that the *cheV* gene does not affect biofilm formation in *SE*.

Drug susceptibility tests were conducted using the K-B method, and the results were interpreted according to the standards of the NCCLS. The results (Table 4) showed that compared to the C50336, Δ*cheV* only exhibited increased resistance to cefoperazone. There were no significant differences in sensitivity to other antibiotics. This indicates that the *cheV* gene has a minimal impact on the drug resistance of *SE*.

**Table 4:** Antibiotic susceptibility test of *cheV* gene deletion strain

Drug Name	Standard of inhibition zone diameter (mm)			Diameter (mm)		
	R	I	S	C50336	ΔcheV	ΔcheV + cheV
Ampicillin	≤ 13	14–16	≥ 17	20	19	0
Piperacillin	≤ 17	18–20	≥ 21	20	18	0
Amoxicillin	≤ 13	14–17	≥ 18	24	22	0
Cefazolin	≤ 19	20–22	≥ 23	24	24	0
Cephalothin	≤ 14	15–17	≥ 18	18	19	0
Cefoperazone	≤ 15	16–20	≥ 21	24	18	22
Cefuroxime	≤ 14	15–17	≥ 18	16	15	20
Ceftriaxone	≤ 13	14–20	≥ 21	17	16	25
Gentamicin	≤ 12	13–14	≥ 15	17	19	27
Amikacin	≤ 14	15–16	≥ 17	18	18	22
Kanamycin	≤ 13	14–17	≥ 18	18	18	26
Streptomycin	≤ 11	12–14	≥ 15	15	15	17
Tetracycline	≤ 14	15–18	≥ 19	15	15	20
Doxycycline	≤ 12	13–15	≥ 16	15	13	16
Minocycline	≤ 14	15–18	≥ 19	11	12	15
Ciprofloxacin	≤ 15	16–20	≥ 21	23	27	27
Levofloxacin	≤ 13	14–16	≥ 17	24	22	30

### The *cheV* gene does not affect the motility of *SE*

**Fig. 3:**
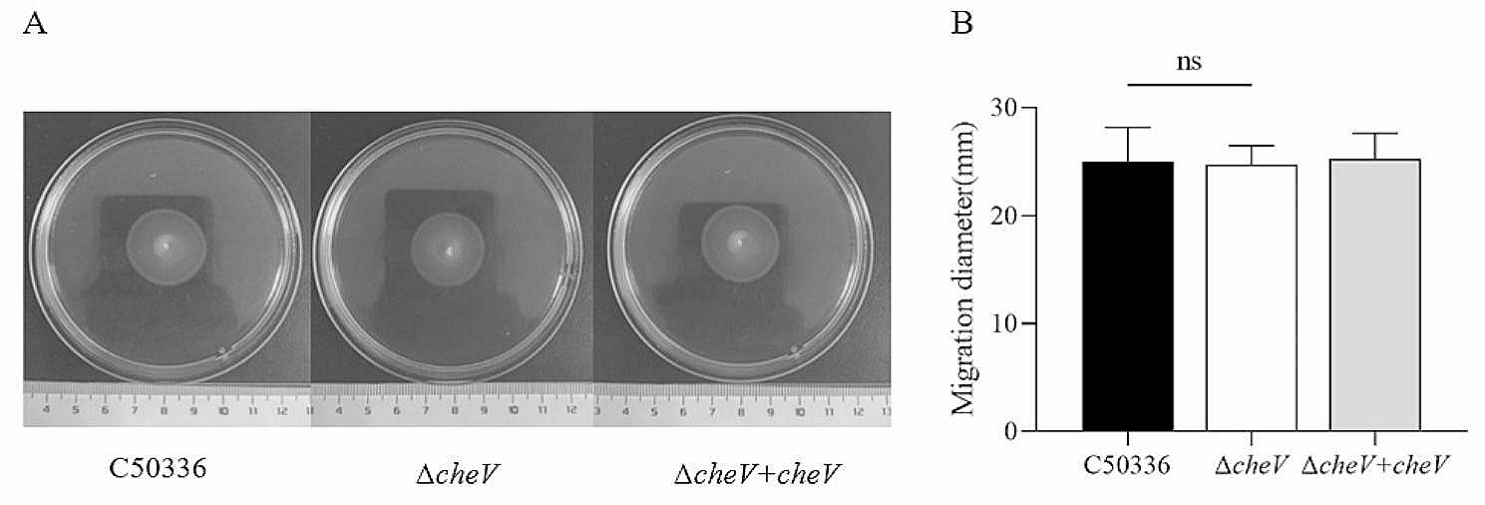
(**A**) The images shown are representatives of several independent assays. (**A**) The motility of the strains was evaluated on 0.3% agar plates, measured after 5 h of incubation. The data represents the average of 3 replicates (ns means not significant)

Using 0.3% semi-solid agar for motility testing, the results showed that the average diameters of motility for the C50336, Δ*cheV* and Δ*cheV* + *cheV* strains were 25 mm, 24 and 25 mm respectively (Fig. 3). There was no significant difference among the 3 strains, indicating that the deletion of the *cheV* gene does not affect the motility of *SE*.

### The *cheV* gene affects the invasion and intracellular survival ability of *SE*

**Fig. 4:**
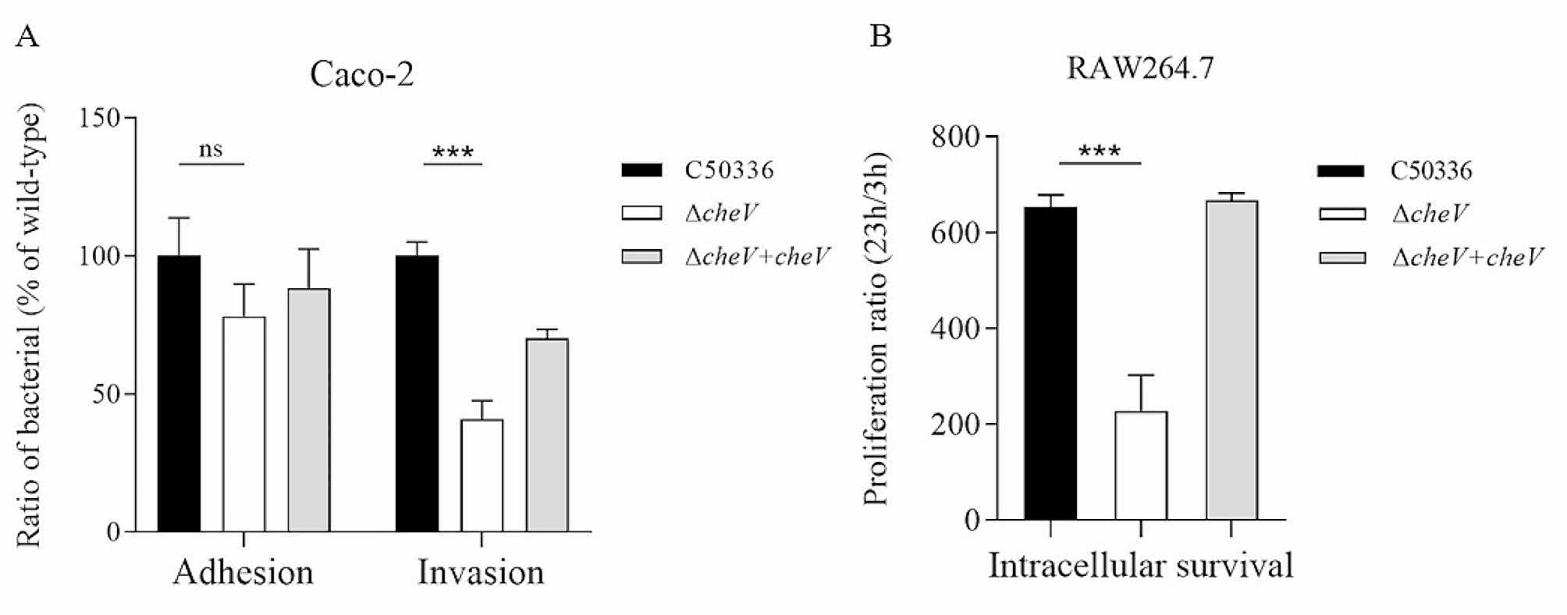
(**A**) Adhesion and invasion of bacteria in Caco-2 cells. (**B**) Intracellular survival in RAW264.7 cells. The results of the adhesion and invasion assays are presented as a ratio to the C50336 (The results of C50336 were considered as 100%). The data represents the average of 3 replicates (***p < 0.001, ns means not significant)

Using Caco-2 and RAW264.7 cell models, the adhesion, invasion and intracellular survival abilities of the Δ*cheV* were tested (Fig. 4). The results showed that the adhesion ability of the Δ*cheV* to Caco-2 cells was not significantly different compared to C50336. However, its invasion ability and intracellular survival ability significantly decreased. These results indicate that the deletion of the *cheV* gene does not affect the adhesion ability of *SE* but does reduce its invasion and intracellular survival capabilities.

### Deletion of the *cheV* gene attenuates the virulence of *SE*

Mice were infected with C50336 and Δ*cheV* via the i.p. route. Both groups began to exhibit mortality on the 3rd dpi, showing typical symptoms such as trembling, arched backs, crusted eyes and disheveled fur. In contrast, the control group mice had smooth fur and good mental state. The LD_50_ calculation results (Table 5) showed that the LD_50_ for C50336 and Δ*cheV* were 6.3 × 10^5^ CFU/mouse and 1.25 × 10^7^ CFU/mouse, respectively. This indicates that the LD_50_ of Δ*cheV* is approximately 20 times higher than that of C50336 (1.25 × 10^7^/6.3 × 10^5^ ≈ 20), suggesting that the deletion of the *cheV* gene reduces the virulence of *SE*.

**Table 5:** LD_50_ of C50336 and Δ*cheV* in KM mice

Strain	Inoculation dose (CFU/mouse)	No. of deaths / total no. of mice	LD_50_ (CFU)	
C50336	2 × 10^7^	5 / 5	6.3 × 10^5^	
	2 × 10^6^	3 / 5		
	2 × 10^5^	2 / 5		
	2 × 10^4^	0 / 5		
	2 × 10^3^	0 / 5		
Δ*cheV*	2.5 × 10^9^	5 / 5	1.25 × 10^7^	
	2.5 × 10^8^	5 / 5		
	2.5 × 10^7^	4 / 5		
	2.5 × 10^6^	0 / 5		
	2.5 × 10^5^	0 / 5		

### Reduced expression levels of virulence genes in *cheV* gene deletion strain

**Fig. 5:**
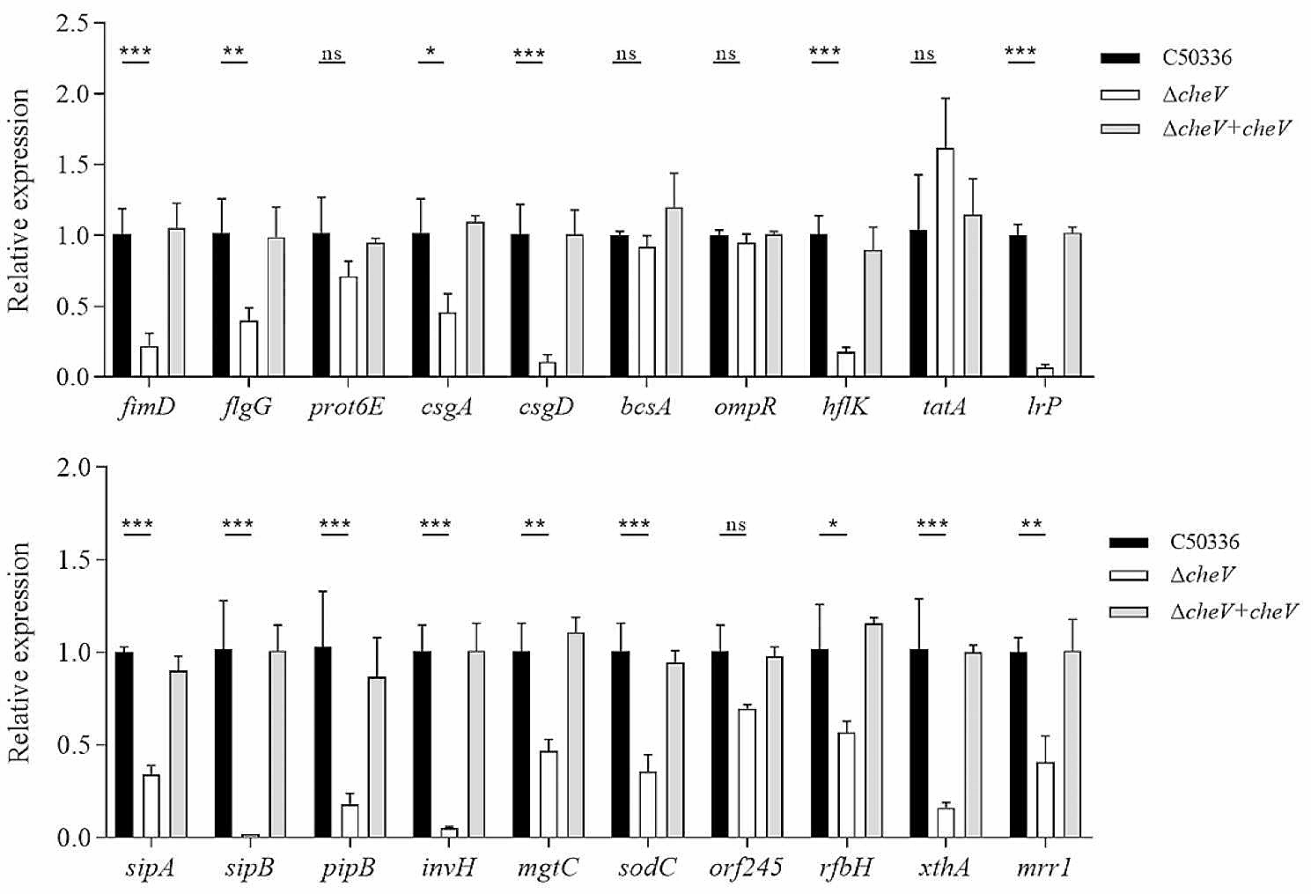
The expression levels of virulence genes in C50336, Δ*cheV* and Δ*cheV + cheV* were detected by using qPCR, with *16 S rRNA* as the housekeeping gene. The data represents the average of 3 replicates (*p < 0.05, **p < 0.01, ***p < 0.001, ns means not significant)

To further investigate the impact of the *cheV* gene on the virulence of *SE*, the expression levels of various virulence genes following the deletion of the *cheV* gene were examined using the qPCR method. The results (Fig. 5) showed significant reductions in the expression levels of *fimD*, *flgG*, *csgA*, *csgD*, *hflK*,*lrp*, *sipA*, *sipB*, *pipB*, *invH*, *mgtC*, *sodC*, *rfbH*, *xthA*, and *mrr1*. This indicates that the deletion of the *cheV* gene leads to reduced expression of multiple virulence genes in *SE*.

### The *cheV* gene deletion strain provides good immune protection in mice

**Fig. 6:**
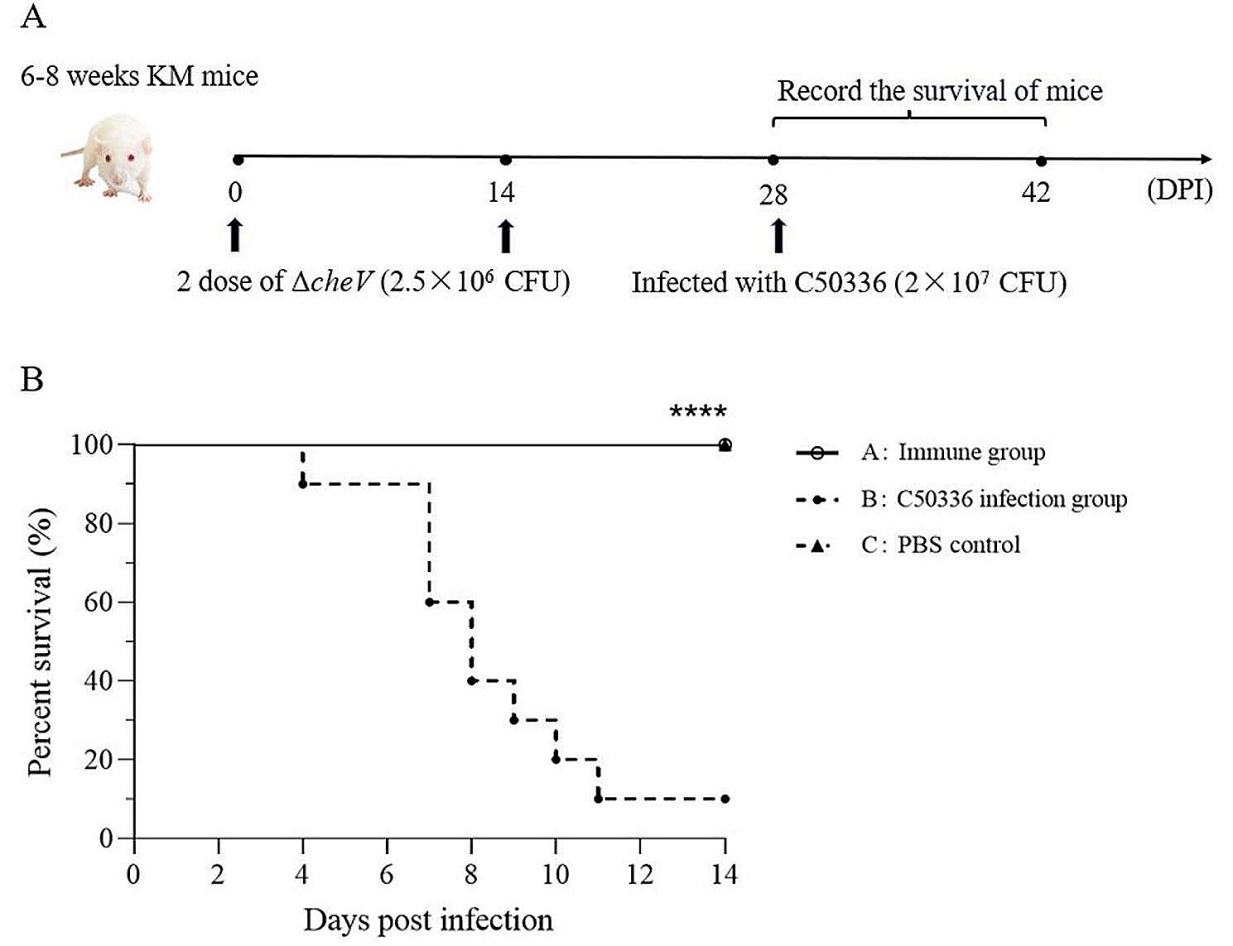
Survival rate of immunized mice against *SE* infection. (**A**) Female KM mice (*n* = 10 per group) aged 6–8 weeks were i.p. injected with the Δ*cheV* and i.p. injected with a lethal dose of C50336 (2 × 10^7^ CFU/mouse) at 28 dpi. The survival rate of mice was monitored daily. (**B**) Survival curve (****p < 0.0001)

14 days after the final immunization, mice in Group A and Group B were simultaneously infected with C50336 through the i.p. route, while Group C was again injected with PBS. The results (Fig. 6) showed that both Group A and Group C mice demonstrated a 100% survival rate over 14 days, with none of the mice exhibiting typical clinical symptoms of *SE* infection. In contrast, the mortality rate in Group B was 90% within the same period, and typical symptoms of *SE* infection, such as trembling, arched backs, crusted eyes and disheveled fur, were observed in these mice. According to the formula for calculating immune protection rate, the immune protection rate of Δ*cheV* is 100%. This indicates that Δ*cheV* provided 100% protection in mice against *SE* infection.

## Discussion

In the process of developing genetically engineered attenuated live vaccines, selecting suitable candidate genes for attenuation is crucial. Chemotaxis affects the survival and colonization abilities of *Salmonella* within the host, which in turn impacts the bacterium’s virulence [31, 33]. The CheV, as a coupling protein in the chemotaxis system, might influence the virulence of *SE*. To confirm this hypothesis, we constructed a *cheV* gene deletion strain. Both in vivo and in vitro assays, it was found that compared to the C50336, the *cheV* gene deletion strain exhibited significantly reduced virulence, indicating that the *cheV* gene plays an important role in the virulence of *SE*.

In the process of pathogenesis, *SE* must contend with many adverse environmental conditions, including antimicrobial peptides, changes in temperature and pH, and limited availability of nutrients [34]. Additionally, the host’s inflammatory response, which recruits phagocytic cells, exposes *Salmonella* to other unfavorable conditions such as oxidative and nitrosative stress [35]. The ability to survive under different environmental stress conditions is a fundamental characteristic of *SE*’s pathogenicity [36]. In this study, we found that the Δ*cheV*, compared to the wild type, showed a significant decrease in survival rate under acid, alkaline, and thermal stress. This suggests that the deletion of the *cheV* gene weakens *SE*’s perception of acid, alkaline, and thermal environments, preventing it from making appropriate responses to avoid and endure stressful conditions, thereby reducing its survival rate and affecting its pathogenic process.

Moreover, biofilm formation is one of *Salmonella*’s adaptive mechanisms for surviving in adverse conditions. It protects the bacteria from harsh external environments, enhancing *SE*’s resistance to conditions such as dryness, extreme temperatures, antibiotics and preservatives [34, 37]. In this study, the biofilm formation ability of *SE* after the deletion of the *cheV* gene was investigated. It was found that there was no significant difference in the biofilm formation of Δ*cheV* compared to C50336, and the production of curli and cellulose was consistent with that of C50336 and Δ*cheV + cheV*. qPCR analysis revealed that the expression levels of the biofilm formation-related genes *csgA* and *csgD* were significantly reduced. A possible explanation is that, although the deletion of the *cheV* gene led to a decrease in the expression levels of some genes related to biofilm formation, biofilm formation is regulated by multiple factors, and overall, the deletion of the *cheV* gene does not significantly affect the biofilm formation capability. Biofilm formation ability is often closely related to bacterial drug resistance [38]. The drug resistance testing of the Δ*cheV* indicated that, compared to C50336, there was no significant change in its sensitivity to most antibiotics, which aligns with expectations. However, it is important to note that the parental strain C50336 is not highly resistant to antibiotics, which might mislead us to believe that the deletion of the *cheV* gene does not affect the bacterial drug resistance.

In the host body, the strength of motility plays a crucial role in whether *Salmonella* can reach specific sites. After entering the small intestine, *Salmonella* must traverse the mucosal layer and then adhere to the intestinal epithelial cells. Therefore, motility is a prerequisite for *Salmonella* to increase its chances of contact with intestinal epithelial cells [39]. Research on different bacteria has found that the impact of *cheV* on motility is inconsistent. In this study, we found that the motility of the Δ*cheV* on semi-solid agar did not change significantly. qPCR analysis showed a decrease in the expression levels of *Salmonella* motility-related genes *flgG* and *fimD*, while the expression level of *prot6E* did not change. Although there is a decrease in the expression levels of genes related to motility, other compensatory mechanisms may exist, leading to the deletion of *cheV* not significantly affecting the motility of *SE*.

To further investigate the impact of the *cheV* gene on the virulence of *SE*, we used both cellular and animal models for evaluating virulence. The pathogenesis of *Salmonella* primarily involves adhesion to epithelial cells, invasion, and survival within macrophages. In this study, we found that deletion of the *cheV* gene did not significantly change the adhesion capability of *SE* to epithelial cells, possibly because the formation of *SE* biofilms and motility were not significantly affected, resulting in no significant difference in the amount of adhesion to epithelial cells. However, the ability to invade and the intracellular survival rate in macrophages significantly decreased. Moreover, qPCR analysis revealed that the expression levels of genes related to invasion ability (*invH*) and genes related to intracellular survival in macrophages (*sipA*, *pipB*, *mgtC* and *sodC*) were significantly reduced. These results suggest that deletion of the *cheV* gene reduces the invasion and intracellular survival abilities of *SE*, leading to attenuated virulence. LD_50_ measurements in mice models for C50336 and Δ*cheV* showed that the LD_50_ of Δ*cheV* increased by about 20 times, indicating that the *cheV* gene influences the virulence of *SE*. To further investigate the role of the *cheV* gene in *SE* virulence, qPCR was used to detect the expression of various virulence genes after *cheV* gene deletion. It was found that the expression levels of genes involved in metabolism (*rfbH*), genes related to exonuclease/endonuclease activity (*xthA* and *mrr1*), genes associated with cell membrane and cell wall integrity (*hflK* and *lrp*), and genes related to type III secretion system (T3SS) (*sipB*, including *sipA*, *pipB*) were significantly reduced. This suggests that the deletion of *cheV* can downregulate the expression of various virulence genes, thereby reducing the virulence of *SE* consistent with the LD_50_ test results. To determine the immune protection rate, mice were immunized twice with Δ*cheV*, and it was found that this method of immunization could 100% resist the infection by the wild-type strain. This result indicates that the *cheV* gene deletion strain has a good immune protective effect on mice and has the potential to be a candidate for developing genetically engineered attenuated live vaccines.

Developing attenuated *Salmonella* live vaccines requires skillfully balancing attenuation with immunogenicity. It’s essential to ensure that the bacterial virulence is appropriately reduced, while also stimulating immunity without causing disease [40]. Therefore, we will next examine the distribution of bacteria in various organs to further assess the virulence level of the Δ*cheV*. Additionally, after immunizing mice, we will measure immune indicators such as IgG, IgA and cytokines to further analyze the immune protection of the mutant.

In conclusion, this study demonstrates that the deletion of the *cheV* gene reduces the virulence of *SE*. and the *cheV* gene deletion strain can provide effective immune protection in mice. These findings lay the groundwork for subsequent research and development of related genetically engineered attenuated live vaccines.

### Electronic supplementary material

Below is the link to the electronic supplementary material.

**Supplementary Material 1**: Figure 1A-S1—The original electrophoretic image of Figure 1 (A)

**Supplementary Material 2**: Figure 1B-S2—The original electrophoretic image of Figure 1 (B)

## Data Availability

The datasets supporting the conclusions of this article are included within the article.
