# Supplementary figures and images for: CheV enhances the virulence of Salmonella Enteritidis, and the Chev-deleted Salmonella vaccine provides immunity in mice
Source: BMC Vet Res. 2024 Mar 11;20:100. doi: 10.1186/s12917-024-03951-x (PMC10926574; doi:10.1186/s12917-024-03951-x)

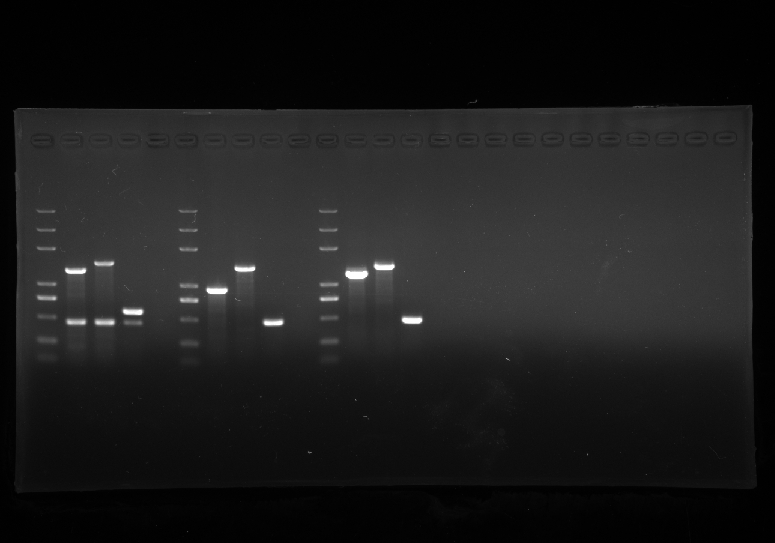

Supplement: Supplementary file 1 — Supplementary Material 1: Figure 1A-S1—The original electrophoretic image of Figure 1 (A) [file 12917_2024_3951_MOESM1_ESM.tif]

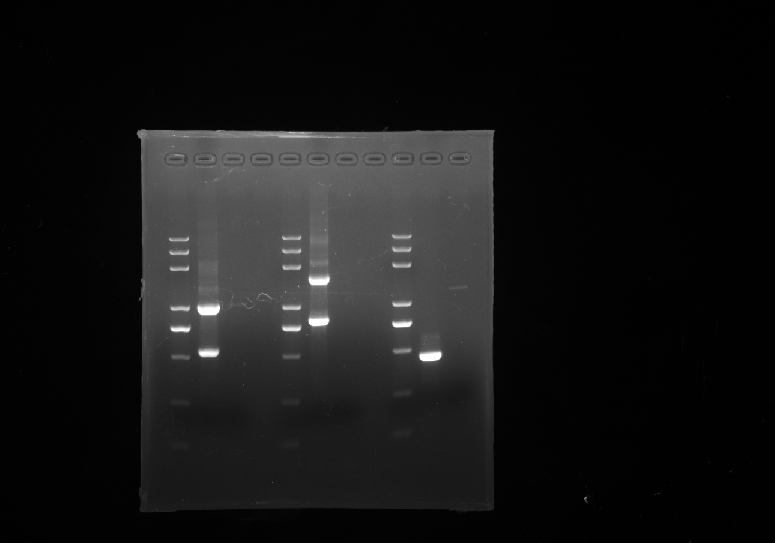

Supplement: Supplementary file 2 — Supplementary Material 2: Figure 1B-S2—The original electrophoretic image of Figure 1 (B) [file 12917_2024_3951_MOESM2_ESM.tif]
